# 1365. Clinical and Financial Implication of Dalbavancin Utilization on Length of Stay Avoidance in Acute Bacterial Skin and Skin Structure Infection

**DOI:** 10.1093/ofid/ofab466.1557

**Published:** 2021-12-04

**Authors:** Sonia S Kim, Brandon Chen, Karan Raja, Mitesh Patel, Mona Philips

**Affiliations:** 1 Clara Maass Medical Center, Belleville, New Jersey; 2 Clara Maass Medical Center, RWJBarnabas Health, Belleville, NJ

## Abstract

**Background:**

Our institution admits 650 patients annually for acute bacterial skin and skin structure infection (ABSSSI). These patients may require intravenous antibiotics, potentially complicated by social factors and loss to follow up. Dalbavancin is a long-acting lipoglycopeptide given as a single dose regimen for ABSSSI. A previous review conducted at our institution identified 117 potential avoidable hospital days over 4 months with outpatient dalbavancin use. The objective of this prospective study was to evaluate the clinical and financial impact of avoided admissions with outpatient dalbavancin use.

**Methods:**

The Institutional Review Board approved this single-site, prospective study. All patients who presented to the emergency department (ED) with ABSSSI from December 15, 2020 to April 15, 2021 were included in the study. Dalbavancin eligibility criteria were given to providers. Eligible patients were given a single dose of dalbavancin and then discharged. The primary outcome was the difference between percentage of avoidable admissions from the ED with dalbavancin use in the retrospective cohort and prospective cohort. The secondary outcomes were estimated length of stay avoidance, percentage of treatment success without ED re-visit within 30 days, estimated hospital cost avoidance and drug cost reimbursement. The primary outcome was assessed using the Chi-square test. Descriptive statistics were used for the secondary outcomes.

**Results:**

Fourteen patients received dalbavancin and avoided hospital admissions. The percentages of admissions avoided in the retrospective and prospective cohorts were 16.02% and 6.67%, respectively (Figure 1). A difference of 9.35% was found to be statistically significant (p=0.01). The total estimated length of stay avoidance was 50 days. No patients re-visited the ED within 30 days with treatment failure. The total estimated hospital cost avoidance was &148,852 (Table 1). The net reimbursement for dalbavancin over drug cost was &5,100 (Table 2).

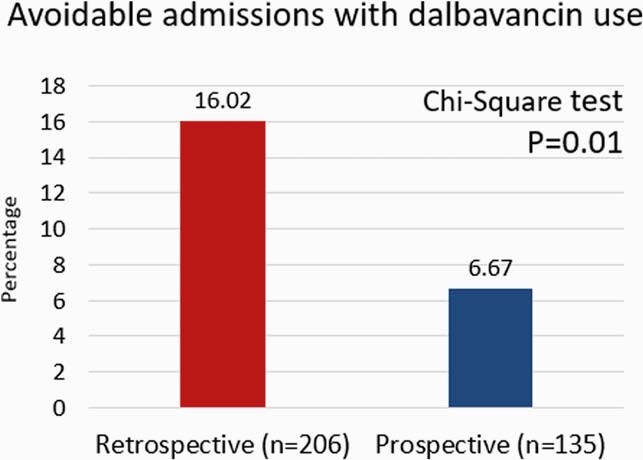

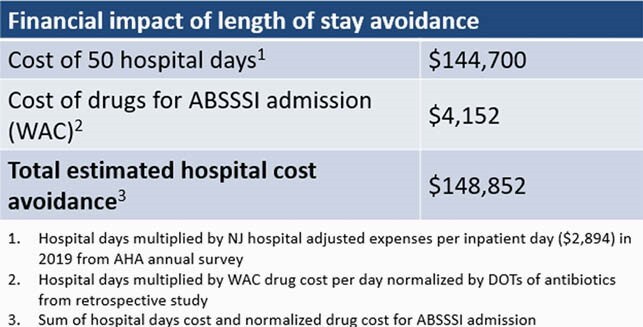

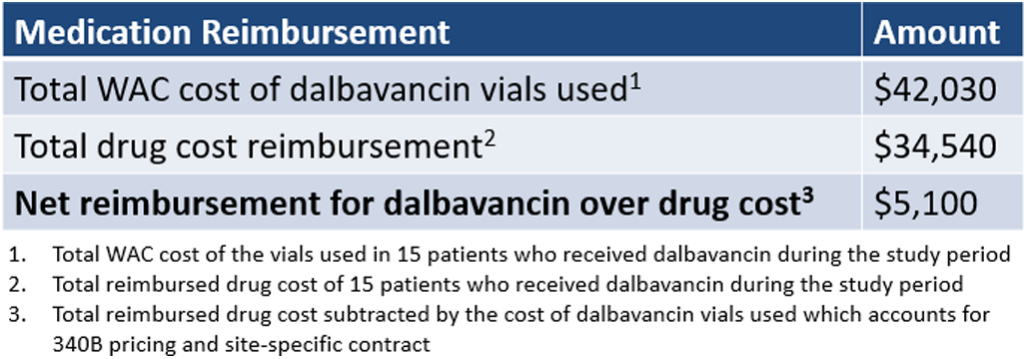

**Conclusion:**

Dalbavancin use decreased avoidable admissions. At our institution, annual hospital cost savings can reach &1,015,794 if dalbavancin was utlilized to all eligible patients.

**Disclosures:**

**All Authors**: No reported disclosures

